# Colonic pedunculated polypoid vascular ectasia mimicking ileocolic intussusception: a rare case report

**DOI:** 10.1097/MS9.0000000000000913

**Published:** 2023-05-26

**Authors:** Ujwal Raut, Santosh Thapa, Garima Shrestha, Sandesh Shah, Utsav Karki

**Affiliations:** a B. P. Koirala Institute of Health Sciences, Dharan; b KIST Medical College and Teaching Hospital, Lalitpur; c Department of Surgery, National Academy of Health Sciences, Kathmandu, Nepal

**Keywords:** Intussusception, polypoid, target sign, vascular ectasia

## Abstract

Vascular ectasias are characterized by abnormal blood vessel enlargement and presumed to be caused by degenerative processes. About 3% of lower gastrointestinal bleeding is caused by it. On endoscopy, colonic arteriovenous malformations are frequently solitary, sizable, flat, or raised red lesions. Conversely, colonic vascular ectasia that manifests as pedunculated polypoid lesions are rare.

**Case presentation:**

A 45-year-old woman presented with hematochezia and abdominal pain. Abdominal ultrasound and Contrast enhanced computed tomography abdomen, both showed features of ileocolic intussusception. Intraoperatively, an intraluminal pedunculated polypoid growth extending up to the hepatic flexure of the colon was discovered. A right hemicolectomy was performed, removing the polypoid growth as well. After histopathological evaluation, a final diagnosis of colonic polypoid vascular ectasia was made.

**Clinical discussion:**

Gastrointestinal bleeding is the common initial manifestation of vascular ectasia, while some individuals may continue to be asymptomatic. According to a study from July 2022, vascular ectasia that manifests as polypoid growth is an uncommon phenomenon that has only been documented in 17 other cases. An intussusception may have a polypoid vascular ectasia as its lead point. Conversely, a large polypoid vascular ectasia may have radiographic characteristics that resemble an intussusception.

**Conclusion:**

Large colonic vascular ectasia, which tends to enlarge over time, can occasionally be misinterpreted as an intussusception due to comparable radiological appearances. In the event that a polypoid colonic vascular ectasia is misidentified for intussusception, the surgical team must be ready to adjust the treatment protocol as needed.

## Introduction

Highlights

- A 45-year-old woman presented with blood-stained stool and right lower abdominal pain.
- The target sign or doughnut sign seen on both the contrast enhanced computed tomography and the ultrasonography led to the presumptive diagnosis of ileocolic intussusception.
- Intraoperatively, intraluminal polypoid growth measuring ~14×2.5×2 cm arose adjacent to the ileocaecal opening which extended up to the hepatic flexure of the large intestine.
- Right hemicolectomy along with end-to-end bowel anastomosis was performed.
- After histopathological evaluation, a final diagnosis of colonic polypoid vascular ectasia was made.

## Background and rationale

Colonic vascular ectasias (VE), commonly referred to as arteriovenous malformations (AVMs) or angiodysplasia, is a disorder characterized by aberrant blood vessel enlargement that is thought to be brought on by degenerative changes^1^. Particularly in the elderly, it is a common cause of lower gastrointestinal bleeding. Usually, they appear as reddish lesions that are flat or slightly elevated. Conversely, colonic VE presenting as pedunculated polypoid lesions are rare^2^ and commonly misidentified for atheroemboli-associated polyps or inflammatory fibroid polyps^3^. The ascending colon or caecum is frequently affected in older patients with vascular ectasia or angiodysplasia, the most common gastrointestinal (GI) vascular malformation^4^. Despite the rarity of colonic vascular ectasia manifesting as a pedunculated polypoid growth, we report a case in a 45-year-old female where a polypoid colonic vascular ectasia mimicked an ileocolic intussusception. The primary learning goal of our shown instance is the potential for such imitating phenomena.

Guidelines: SCARE 2020 paper^5,^ Supplemental Digital Content 1, http://links.lww.com/MS9/A143.

This case has been reported in line with the SCARE criteria.

## Case report

### Patient information

#### Demographics and presentation

A 45-year-old female presented to the emergency with complaints of blood-stained stool for 1 day. The patient was apparently well until 7 days back when she developed pain over the right lower abdomen. The pain was colicky in nature, moderate intensity, and non-radiating, with no aggravating or relieving factors. The pain was associated with vomiting for 2 days which was non-bilious, occurred two to three times per day, and contained only food particles. On further inquiry, the patient reported having constipation for 6 months which occurred in an on-and-off pattern. She did not report any pain during defecation nor any protruding mass per rectum. There was no history of melena, anorexia, weight loss, or fever.

#### Past medical and surgical history

No significant past medical and surgical history.

#### Family history

No family history of similar presentations exists.

#### Drug and allergy history

No significant drug and allergy history. The patient denied the use of tobacco or illicit drugs.

## Clinical findings

On examination, the patient was alert with stable vital signs. The abdomen was soft with tenderness present over the right iliac fossa. No guarding, rigidity, or rebound tenderness was noted. No organomegaly or lump was palpable. The bowel sounds were increased. Hernial orifices were intact. Blood-stained faeces were found during a Digital Rectal Examination (DRE). DRE was normal in all other aspects. The rest of the general and systemic examinations were within normal limits.

## Diagnostic assessment and interpretation

**Figure 1:**
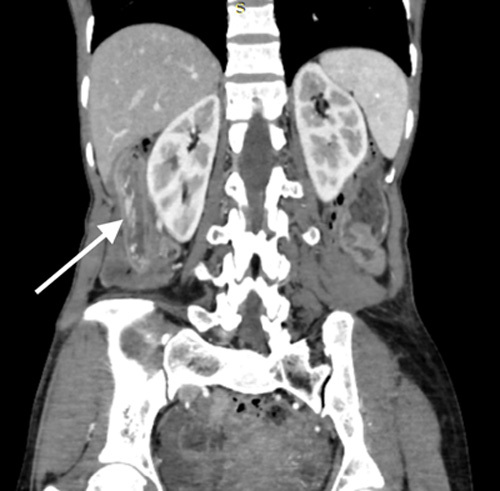
Contrast enhanced computed tomography (CECT) of the abdomen and pelvis in coronal reformation shows features of telescoping of distal ileum into caecum and ascending colon with an intussusceptum length of 13 cm (white arrow).

**Figure 2:**
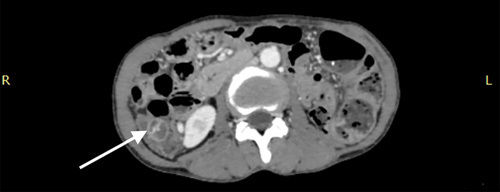
Contrast enhanced computed tomography (CECT) of the abdomen and pelvis in axial reformation shows “target sign”(white arrow).

Laboratory studies showed haemoglobin levels of 8.6 gm/dl. Other blood investigations were unremarkable. Abdominal ultrasonography revealed a “target sign”, possibly due to the telescoping of the terminal part of the ileum into the caecum. The patient underwent a contrast enhanced computed tomography abdomen which revealed features suggestive of telescoping of the distal ileum into the caecum and ascending colon, measuring a total intussusception length of 13 cm (Figs. 1 and 2, SDC video 1, Supplemental Digital Content 2, http://links.lww.com/MS9/A144). A provisional diagnosis of ileocolic intussusception was made.

## Intervention

**Figure 3:**
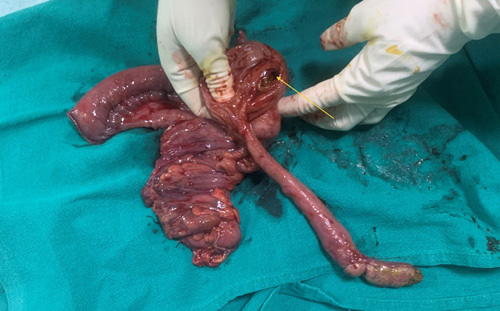
Right hemicolectomy specimen which includes pedunculated polypoid growth measuring ~15 × 2 cm arising adjacent to the ileocecal opening (yellow arrow).

**Figure 4:**
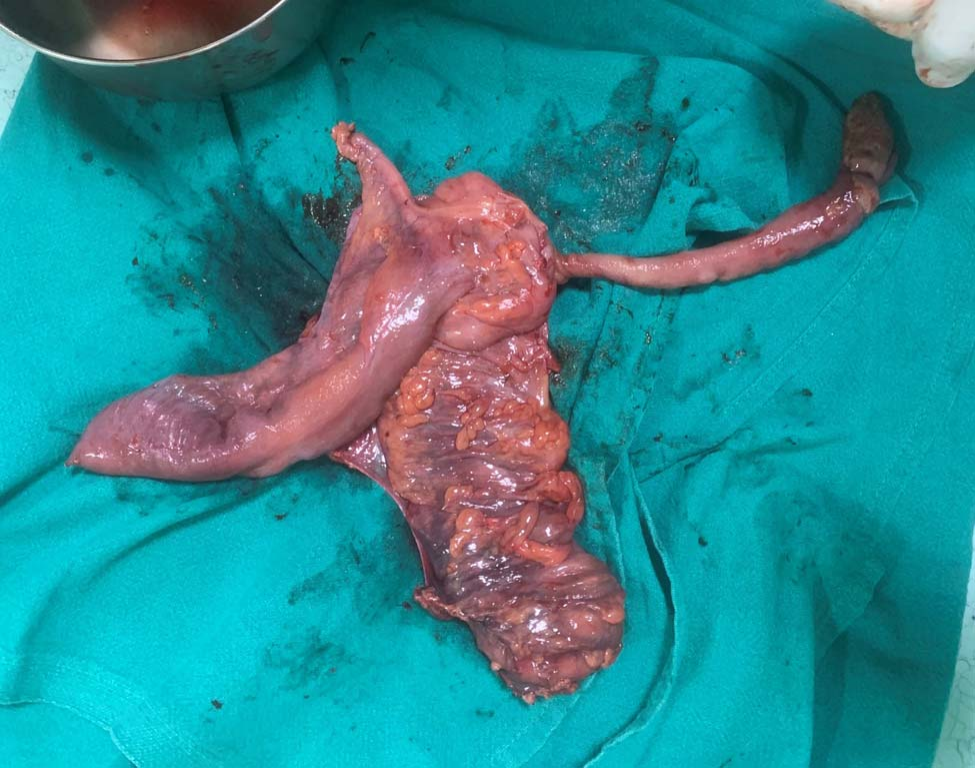
Right hemicolectomy specimen which includes pedunculated polypoid growth measuring ~15 × 2 cm.

The decision to operate on the patient was validated by the imaging findings. Intraoperatively, the terminal ileum and caecum looked normal with no telescoping of the bowel loops. An intraluminal mass with firm consistency was palpated. On further exploration, an intraluminal polypoid growth measuring ~14×2.5×2 cm arose adjacent to the ileocaecal opening which extended up to the hepatic flexure of the large intestine (Figs. 3 and 4). Right hemicolectomy with end-to-end bowel anastomosis was performed with consent after taking into account the patient’s financial circumstances and follow-up compliance. The sample was sent for histopathologic examination. The patient and her family were counselled regarding the intraoperative finding. The patient was admitted to the postoperative ward with IV antibiotics, pain medication, and fluids. Soft diets were introduced gradually and she was discharged on 4th postoperative day without complications.

## Follow-up and outcome

**Figure 5:**
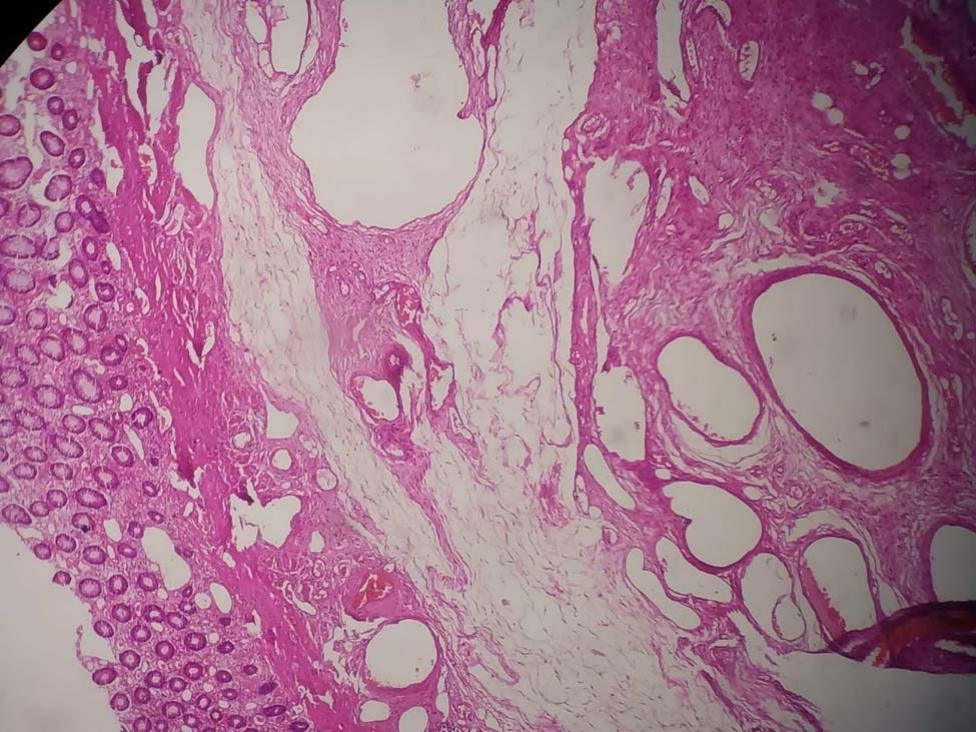
Numerous intermediate to large-sized blood vessels with thin walls are seen extending from submucosa to muscularis propria along with fibroblast proliferation.

**Figure 6:**
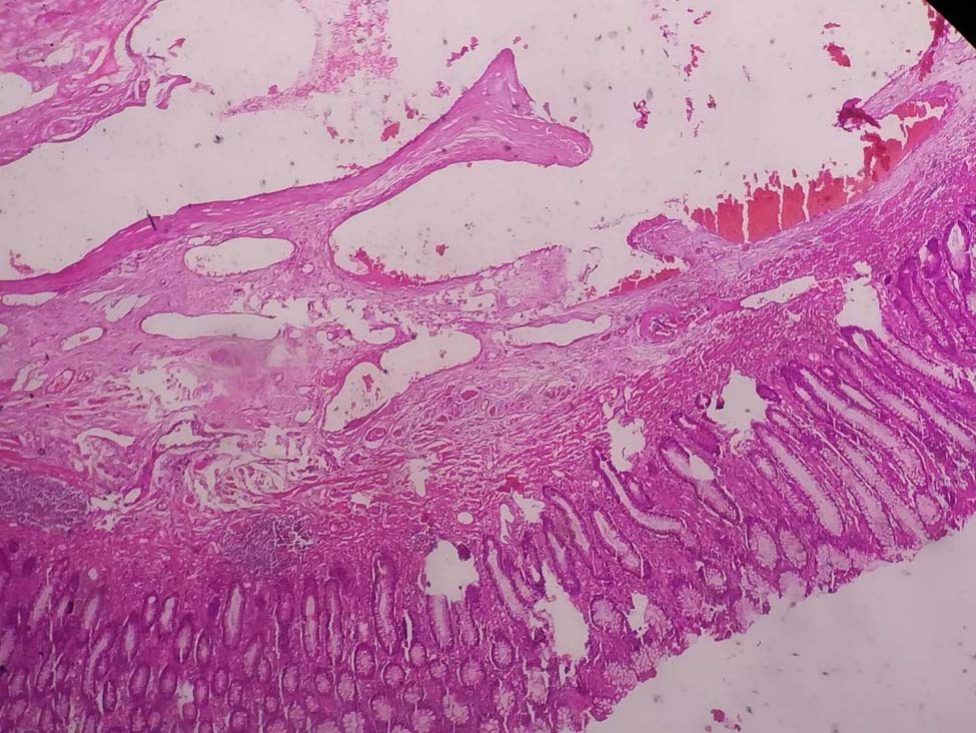
Colonic crypts and glands lined by columnar cells along with interspersed goblet cells. Numerous intermediate to large-sized blood vessels with thin walls are seen extending from submucosa to muscularis propria along with fibroblast proliferation.

The patient followed up 2 weeks later with the histopathology report which showed vascular ectasia (Figs. 5 and 6). She was tolerating a low-fibre diet and her pain was well controlled. She had a return of normal bowel movements with no per rectal bleeding. The surgical incisional site was healthy and the Eastern Cooperative Oncology Group (ECOG) Performance Score was “1”. The patient was advised to undergo a screening colonoscopy at 6 months.

## Discussion

VEs are a type of acquired AVM that differs from neoplastic and congenital lesions. They can occasionally affect younger persons but more frequently affect elderly patients as the cause of occult, recurrent, or severe colonic haemorrhage^6^. According to their morphology, VE is made up of dilated, thin-walled vessels in the mucosa and submucosa that are lined entirely or mostly by endothelium along with a small amount of smooth muscle^7,8^. The leading hypothesis is that these lesions grow with age as a result of intermittent and recurrent mucosal and submucosal vein occlusion brought on by increased contractility at the level of the muscularis propria. The prolonged obstruction causes vascular dilatation and arteriovenous shunts by increasing pressure and congestion in venules and capillaries^9,10^.

Clinic visits and hospital stays are frequently caused by GI bleeding from the colon. About 3% of lower GI bleeding is caused by vascular ectasias^3^. Hematochezia is the primary concern in 60% of patients with colonic AVMs, while 25% of the lesions are asymptomatic in the elderly and discovered during a screening colonoscopy^4^. Similar to our patient, the main reason for the hospital visit was hematochezia. Her haemoglobin was 8.6 gm/dl at evaluation. She had, however, never undergone a screening colonoscopy before. The incidence of vascular ectasia is increased in people with comorbid conditions such as aortic stenosis, Von Willebrand disease, and chronic kidney illness^11^.

Submucosal vascular ectasias presenting as pedunculated polypoid lesions, which are uncommon and have only been described in 17 other cases in the literature, are different from flat or slightly raised classical vascular ectasias, which are reported in 1–6% of colonoscopies, according to a study from July 2022^2^. In our case, the vascular ectasia presented as a pedunculated polypoid growth measuring a size 14×2.5×2 cm. The vascular ectasia’s polypoid growth presentation turned out to be a rare occurrence in and of itself, and the case’s rarity was further enhanced by the growth’s dimensions. The growth originated near the ileocecal opening and extended up to the colon’s hepatic flexure, resembling an ileocolic intussusception on radiographic imaging. According to a thorough assessment of the literature and to the best of our knowledge, the current case appears to be the largest polypoid vascular ectasia ever documented.

When one intestinal segment telescopes into an adjacent intestinal segment, it is called an intussusception^12,13^. In contrast to intussusception in children, intussusception in adults is exceedingly rare, and the majority of cases are secondary^14^. Imaging modalities are necessary for a firm diagnosis. The target or doughnut sign on an ultrasound verifies the diagnosis. The hyperechoic central core of the bowel and mesentery is surrounded by the hypoechoic outer oedematous bowel, creating a doughnut-shaped image on transverse sonography or computed tomography. Intussusception may look like a sandwich when viewed in longitudinal imaging^15^. An intussusception may have a polypoid vascular ectasia as its lead point^16^. In our situation, the axial reformation of the contrast enhanced computed tomography as well as the ultrasonography both showed the target sign or doughnut sign, leading to a presumptive diagnosis of ileocolic intussusception. The likelihood of this imitating phenomenon may result from the intussusception’s common site of occurrence as well as from the polypoid vascular ectasia’s thin-walled tubular structure.

In previous studies, an endoscopic snare polypectomy was mostly used to remove the polypoid vascular ectasia^2^. But, in our situation, the pertinent history, examination, and radiographic evidence of ileocolic intussusception led to a decision to perform laparotomy, only to discover normal bowel walls devoid of any telescoping, as well as a large intraluminal pedunculated polypoid growth for which bowel resection was done along with the polypoid growth.

Both from the patient’s and the physician’s perspective, challenges, difficulties, and limitations were noted. Due to the typical radiographic findings and clinical manifestations, we were unable to appropriately diagnose this case preoperatively. A rapid pathological investigation would have changed the care plan, but our surgical setting did not permit it. Financial obstacles and follow-up compliance issues, such as the patients’ remote residential location, were present from the patient’s perspective.

## Conclusion

Vascular ectasias are caused by degenerative processes, and they may enlarge as the patient ages. Colonic vascular ectasia that manifests as pedunculated polypoid growth is a very uncommon occurrence. A large colonic vascular ectasia could mimic an intussusception, both clinically as well as radiographically. When a surgeon operates on an adult patient with a diagnosis of ileocolic intussusception, even though it is a rare occurrence, this possibility may need to be kept in mind to make the required preoperative and intraoperative treatment modifications.

## Ethical approval

None.

## Informed consent

Written informed consent was obtained from the patient for the publication of this case report and accompanying images. A copy of the written consent is available for review by the Editor-in-Chief of this journal on request.

## Source of funding

This case has not been funded by any person or any institution.

## Author contribution

U.R.: conceptualization, methodology, writing—original draft preparation, software, project administration. S.T.: data curation, writing—original draft preparation, software. G.S.: visualization, writing—reviewing and editing. S.S.: supervision, visualization: U.K.: validation, supervision, conceptualization.

## Conflicts of interest disclosure

None.

## Guarantor

Utsav Karki.

## Data availability statement

NA.

## Provenance and peer review

Not commissioned, externally peer-reviewed.

## Supplementary Material
